# Molecular origin of contact line stick-slip motion during droplet evaporation

**DOI:** 10.1038/srep17521

**Published:** 2015-12-02

**Authors:** FengChao Wang, HengAn Wu

**Affiliations:** 1 CAS Key Laboratory of Mechanical Behavior and Design of Materials, Department of Modern Mechanics, University of Science and Technology of China, Hefei, Anhui 230027 People’s Republic of China

## Abstract

Understanding and controlling the motion of the contact line is of critical importance for surface science studies as well as many industrial engineering applications. In this work, we elucidate the molecular origin of contact line stick-slip motion during the evaporation of liquid droplets on flexible nano-pillared surfaces using molecular dynamics simulations. We demonstrate that the evaporation-induced stick-slip motion of the contact line is a consequence of competition between pinning and depinning forces. Furthermore, the tangential force exerted by the pillared substrate on the contact line was observed to have a sawtooth-like oscillation. Our analysis also establishes that variations in the pinning force are accomplished through the self-adaptation of solid-liquid intermolecular distances, especially for liquid molecules sitting directly on top of the solid pillar. Consistent with our theoretical analysis, molecular dynamics simulations also show that the maximum pinning force is quantitatively related to both solid-liquid adhesion strength and liquid-vapor surface tension. These observations provide a fundamental understanding of contact line stick-slip motion on pillared substrates and also give insight into the microscopic interpretations of contact angle hysteresis, wetting transitions and dynamic spreading.

Motion of the contact line has been a long-standing subject of intense interest^1^,^2^,^3^,^4^, not only for the basic research on the ubiquitous nature^5^,^6^,^7^,^8^, but also because of its important implications across a myriad of related applications^9^,^10^,^11^. Within the framework of continuum hydrodynamics and no-slip condition, an infinite viscous force is derived to move the contact line^12^,^13^. This well-known singularity at the contact line implies that the contact line should always remain pinned on the solid substrate, which is apparently far from the reality. Various mechanisms have been proposed to account for the moving contact line paradox, such as the slip boundary condition^14^, the diffuse interface layer^15^ and the microscopic precursor film^16^, which indicate that there are still considerable challenges in understanding the motion of the contact line^17^,^18^.

Recently, the stick-slip motion of the contact line has been evidenced and widely discussed^19^,^20^,^21^. Alternating pinning and depinning phenomena at the contact line have been more obviously observed during evaporation of liquid droplets on the pillared substrates^22^,^23^,^24^,^25^. It has also been determined that the pinning-depinning transitions at the contact line result in different evaporation regimes^26^,^27^,^28^,^29^. This fact raises several essential questions, such as how to characterize these forces being exerted on the contact line from a microscopic perspective, and also at what point the contact line begins to move. When dealing with questions about wetting on heterogeneous surfaces as shown in Fig. 1(a), Cassie-Baxter’s law is always used^30^,


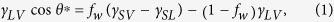


Here *γ*_*LV*_, *γ*_*SV*_ and *γ*_*SL*_ are liquid-vapor, solid-vapor and solid-liquid interfacial tensions, respectively. θ^*^ is the apparent contact angle and *f*_*W*_ represents the surface fraction of the pillars. However, Cassie-Baxter's law is lack of capacity to describe whether the contact line can move or not. In addition, it can not provide an explicit interpretation of the forces exerted on the contact line. The pinning effect of solid is separated into *γ*_*SL*_ and *γ*_*SV*_ thermodynamically, and experimentally measuring the surface tension of solid is still an open challenge^5^.

From a microscopic perspective, the forces acting on the contact line can be classified into two different types, (i) the liquid-liquid interaction force *f*_*L*_ which pulls the contact line towards the interior of the droplet; and (ii) the solid-liquid interaction force *f*_*S*_ which works to make the contact line stay on the substrate. The *f*_*L*_ exerted on the contact line by other portions of the liquid is mainly contributed by the liquid-liquid interactions localized at the interface^31^,^32^. These interactions include *f*_*1*_ parallel to the liquid-vapor interface and *f*_*2*_ along the solid-liquid interface, as shown in Fig. 1(b). For droplets in an equilibrium state with an apparent contact angle θ^*^, it has been shown that the tangential force per unit length exerted by the surrounding liquid on the contact line is 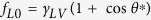
32,33. In this situation, 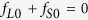 and so the contact line would not move.

The stick-slip motion of the contact line implies that there is competition between the pinning and depinning forces acting on the contact line. Previous work consistently used the contact angle hysteresis force, 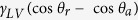 in which *θ*_*a*_ and *θ*_*r*_ are the advancing and receding contact angles, respectively, to quantify the maximum pinning force that the solid substrate could provide^2^,^28^,^34^. In actuality, both the pinning and depinning forces vary instantaneously with the dynamic contact angle. Though many studies have accumulated over understanding and quantifying of the contact line force, including elegant models^32^,^33^ and ingenious experiments^35^,^36^, the fundamental mechanisms underlying the stick-slip motion of the contact line are not yet clear and therefore remain a topic of interest. In this work, we demonstrated a molecular dynamics (MD) based strategy to analyze the molecular origin of the stick-slip motion of the contact line during evaporation. The analysis of forces at the contact line was carried out through directly calculating the molecular interactions. Though the pinning forces acting on the contact line are the major focus for assessment, it should be emphasized that the contact line is an imaginary line that does not represent actual material^31^. Therefore, to be specific, this work considers the forces exerted on the liquid corner in the vicinity of the contact line, which is referred to as the forces acting on the contact line for the sake of simplicity. To the best of our knowledge, the pinning force exerted during the evaporation process has not been explicitly calculated or interpreted in any other work.

## Results and Discussion

### Evaporation-induced stick-slip motion of contact line

**Figure 2:**
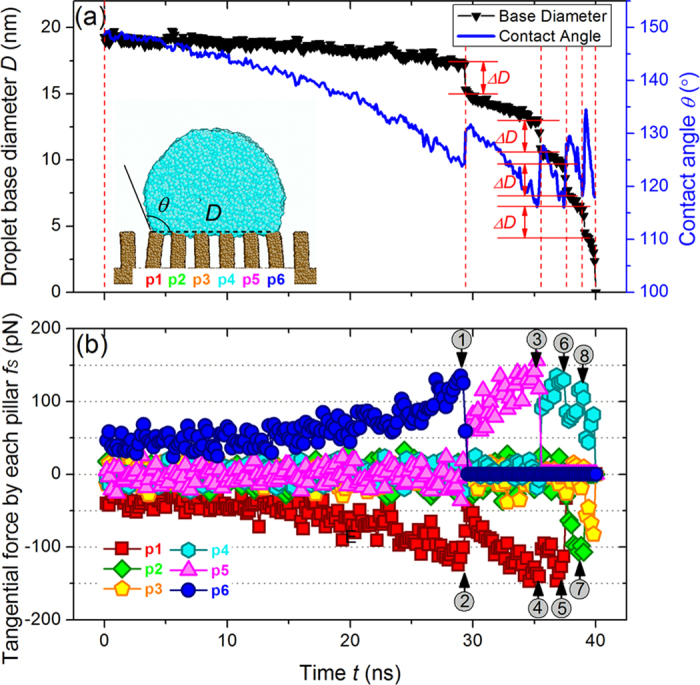
(**a**) Variations in both the base diameter and the contact angle of the droplet during a typical evaporation-induced stick-slip motion (*α* = 1.5, *β* = 0.4, *χ* = 20). The evaporation process is divided into five stages by vertical dash lines, each stage indicating a moment that the contact line was pinned onto the substrate. The inset MD snapshot shows the droplet shape just prior to the first slip event occurs, and demonstrates droplet contact with 6 pillars, p1–p6. (**b**) The tangential forces exerted by each pillar, p1–p6, on the contact line during evaporation. Each abrupt force attenuation has been numbered, which odd numbers indicating the slip events of the contact line.

Using techniques present below in the Methods section, evaporation-induced stick-slip motion of contact line on pillared surfaces was successfully simulated, as shown in Fig. 2(a). The base diameter *D* as well as the instantaneous contact angle *θ* of the liquid droplet were calculated during evaporation and the resulting values did indeed confirm that the contact line experiences a stick-slip motion. As can be seen in Fig. 2(a), this typical evaporation process is divided into five stages, each of which indicates a moment that the contact line is pinned onto the substrate. The stick stage is followed by a slip (or jump) event of the contact line motion in which the contact line retreats from one pillar and jumps to the nearest neighboring pillar. For liquid droplet evaporation on chemically heterogeneous surfaces, slip and jump can be clearly distinguished^29^. However, this process occurs in a very short time period in our MD simulations. Thus we named here the stick-slip motion of the contact line just simply following the conventions in the literatures^19^,^20^,^21^. During each stick stage, the two outer pillars exert tangential forces on the liquid droplet that work to pin the contact line. The reaction forces applied on the substrate lead to the bending deformation of these outer pillars towards the interior of the liquid^37^, as depicted in the inset of Fig. 2(a).

Furthermore, we found that the contact angle *θ* gradually decreases in each stick stage as the liquid evaporating. Due to the unbalanced surface tension, an additional depinning force, 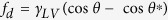 (per unit length), acts on the contact line^24^,^26^. Horizontal component of this depinning force points towards the interior of the liquid droplet. Because the contact line remains static, the pinning force *f*_*p*_ emerges, and is defined as the additional force exerted by the solid substrate in opposition to the depinning force. The balanced condition of the horizontal force acting on the contact line is described as 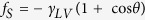, or


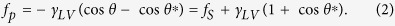


The continuous decrease of *θ* in each stick stage leads to an increase in the depinning force *f*_*d*_ and leads to further bending of the two outer pillars. Due to this effect, it was observed that the base diameter of the droplet decreased slightly during each stick stage, as shown in Fig. 2(a). Further observations show that the contact line is still pinned on the outermost edge of the outer pillar in the stick stage.

**Figure 3:**
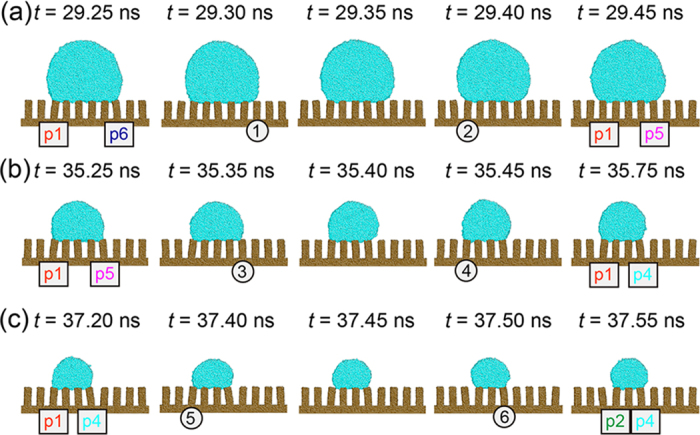
Time-dependent snapshots of a simulated droplet evaporating on a flexible nano-pillared substrate during the first (**a**), second (**b**) and third (**c**) stick-slip events, respectively. Circled numbers denote abrupt force attenuation due to either contact line retreat or droplet shape relaxation, which are corresponding to that in Fig. 2(b). Snapshots were processed using AtomEye^47^.

From equation (2), it can be seen that during evaporation the magnitude of pinning force on the contact line varies in conjunction with the instantaneous contact angle *θ*. During evaporation simulations, we calculated the tangential force exerted on the liquid droplet by each solid pillar, *f*_*S*_, which show a saw-tooth pattern, as illustrated in Fig. 2(b). In order to understand more precisely the way in which the contact line retreats from one pillar and jumps to the nearest neighboring pillar, MD snapshots were recorded for the first three stick-slip events and shown in Fig. 3. By examining Fig. 2(b) and Fig. 3 together, it is possible to interpret each abrupt force attenuation on the atomic scale. Initially, the contact line is pinned on pillars p1 and p6. The initial values of *f*_*S*_ for p1 and p6 indicate the magnitude of *f*_*So*_, as shown in Fig. 2(b). In our MD simulations, this initial value of the tangential force from p1 is about –(32.0 ± 9.5) pN, which is averaged in a time interval of 1.0 ns. The force *f*_*S*_ exerted by these two pillars increases as the contact angle decreases. In the cases of the other pillars, the magnitude of *f*_*S*_ during the first stick stage is near zero, oscillating slightly due to the thermal fluctuations. At about *t *= 29.30 ns, the first stick-slip event occurs and the contact line on the right side retreats to p5, losing contact with p6. As would be expected, the tangential force imposed by p6 on the contact line then fell abruptly to zero, as shown in Fig. 2(b). The base diameter of the droplet also decreased, *ΔD *≈ 2.4 nm, in correspondence with the pillar periodicity *d* minus the deflection of the bending pillars. The first stick-slip event is numbered as 1 in both Figs 2(b) and 3(a). The point numbered 2 in Figs 2(b) and 3(a) shows that although the contact line on the left side remains pinned at p1, the tangential force imposed by p1 decreases abruptly. This is because after the first stick-slip event, the droplet reshapes itself, including an increase in the contact angle. Consequently, the depinning force on the contact line decreases, so does the magnitude of *f*_*S*_. Then the tangential force imposed by p1 increases from a value of about –(60.0 ± 14.1) pN in the next stick stage The tangential force evolution for subsequent stick-slip events can be analyzed in a similar way. These results are generally consistent with 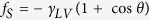. Nevertheless, it should be noticed that even the tangential force at each point provided in Fig. 2(b) was calculated by averaging 200000 data sets, the results still show significant fluctuations. For the droplet evaporation on the pillared substrate shown in Fig. 2, θ^*^ was calculated to be 147.8° ± 3.3°. When the droplet is on the homogeneous flat surface with the same interaction parameters, the corresponding equilibrium contact angle 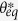 is 122.3° ± 4.8°. Thus we have 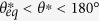. In consideration of the fluctuation in contact angle and the statistical uncertainty during evaporation on the pillared surfaces, 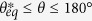 is still hold and 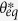 can be used to compare with the lowest value of the instantaneous contact angle in Fig. 2(a), 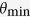 = 118.2° ± 2.1°.

### Pinning force analysis

**Figure 4:**
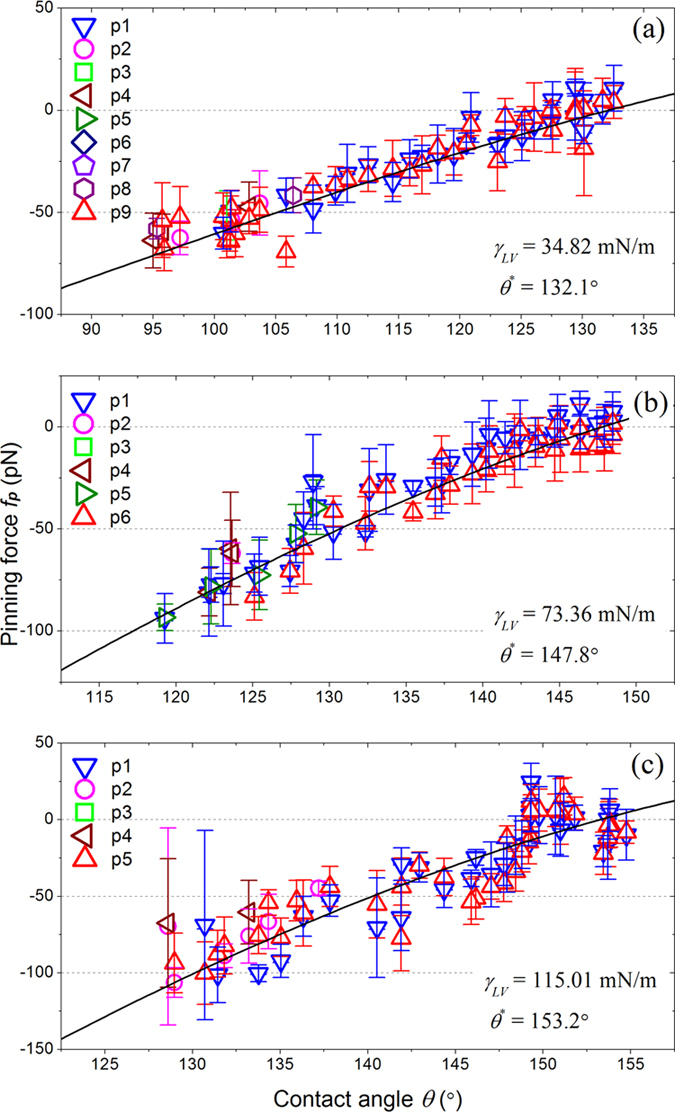
Variation in pinning force exerted on the contact line by each pillar plotted as a function of the contact angle. (**a**) *γ*_*LV*_ = 34.82 mN/m, (**b**) *γ*_*LV*_ = 73.36 mN/m, and (**c**) *γ*_*LV*_ = 115.01 mN/m. The pinning force is directed towards the outward of the droplet, defined as the negative direction. The solid lines plotted in each panel show the fit of equation (2). The error bars show the standard deviations.

As illustrated by equation (2), the pinning force is determined both by *γ*_*LV*_ and by the difference between the instantaneous contact angle *θ* and the apparent contact angle θ^*^. In order to verify the applicability of equation (2), parallel evaporation simulations were run using different *γ*_*LV*_ values generated by varying *ε*_*LL*_, then the variations in *f*_*p*_ exerted by each pillar on the contact line were plotted as a function of *θ*, as shown in Fig. 4. Independent MD simulations were run using a planar liquid film to calculate *γ*_*LV*_^38^,^39^. *ε*_*LL*_ was chosen to equal *k*_*B*_*T*, 1.5 *k*_*B*_*T*, and 2 *k*_*B*_*T*, and *γ*_*LV*_ values were calculated to be 34.82, 73.36 and 115.01  mN/m, respectively. When compared with the MD results, equation (2) was multiplied by the length of contact line, *i.e.*, the thickness of the simulation box, 10*a*. Thus *f*_*p*_ has a unit of pN. The tangential force exerted by the solid on the droplet is only restricted in the vicinity of the contact line. Far from the contact line, this force is zero due to the symmetry^40^. Consequently, only the liquid molecules within the liquid corner near the contact line contribute to *f*_*p*_. Thus it is reasonable to calculate these forces using the definition of the contact line presented in the Methods section. According to equation (2), the apparent contact angle θ^*^ is important in the calculation of *f*_*p*_. θ^*^ may change when the contact line shrinks along the top of a single pillar^29^,^41^. However, in this slip (jump) stage, the pinning force imposed by the solid pillar on the contact line fell abruptly to zero. Moreover, the position of contact line and the contact angle in this stage are difficult to be defined. Only the values of the pinning force in each stick stage were used to compare with the analytical equation. For these reasons, the apparent contact angle θ^*^ used to calculate the pinning and depinning forces in the equation (2) is assumed to be constant in the present work. As can be seen in Fig. 4, equation (2) provides a good fit for the simulation results of *f*_*p*_, as we initially anticipated. These results demonstrated that equation (2) is a good model for assessing the pinning force, not only for static wetting^32^, but also for quasi-static droplet evaporation scenarios.

These MD results demonstrate that in each stick stage of the evaporation, the pinning force is not constant. It actually continuously adjusts to balance the variations in the depinning force. Once the depinning force exceed the maximum pinning force that the solid pillar can provide, the contact line would detach from the pillar and retreat to the next one. This behavior constitutes the molecular origin of contact line stick-slip motion.

The pinning force originates from solid-liquid intermolecular interactions between the solid pillar and the molecules within the within the liquid corner in the vicinity of the contact line, which are described by the Lennard-Jones (LJ) potential, equation (5) in the Methods section. This interaction force between a pillar and the contact line can be written as 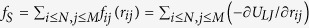, where *r*_*ij*_ is the intermolecular distance between solid atom *i* and liquid atom *j*. *N* and *M* are the total number of solid or liquid atoms contributing to the interactions, respectively. For certain solid and liquid, it can be inferred that the magnitude of *f*_*p*_ is mainly effected by the solid-liquid intermolecular distance *r*_*ij*_ and the number of liquid (or solid) molecules that experience this force. During every stick stage of the evaporation, molecules in the liquid corner gradually vanish, leading to a decrease of *θ*. However, MD results clearly show that the magnitude of *f*_*p*_ increases in each sequential stick stage. This indicates that the number of liquid molecules experiencing pinning forces is not dominant in determining the magnitude of *f*_*p*_. Then we turned to consider the influence of solid-liquid intermolecular distance on *f*_*p*_. For the liquid molecules, the magnitude of thermal fluctuations is comparable to the magnitude of potential energy, 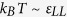 and both internal attractive and repulsive forces coexist in the liquid state^32^. Thus the individual atom-pair interactions are strongly dependent on *r*_*ij*_. From a microscopic perspective, the liquid molecules in the vicinity of the contact line could dynamically adjust their positions in order to cope with variations in the depinning force. A consistent interpretation of these facts is that the pinning force varies instantaneously by adapting the solid-liquid intermolecular distance in order to balance the depinning force. Deformation of the pillar might also contribute to the variation of solid-liquid intermolecular distance. It is possible that this finding could be extended in order to interpret the molecular origin of the contact line stick-slip motion not only for the pillared surfaces but also for smooth or rough surfaces. A detailed explanation of this interpretation is given in following subsection.

### Microscopic origins of pinning force

**Figure 5:**
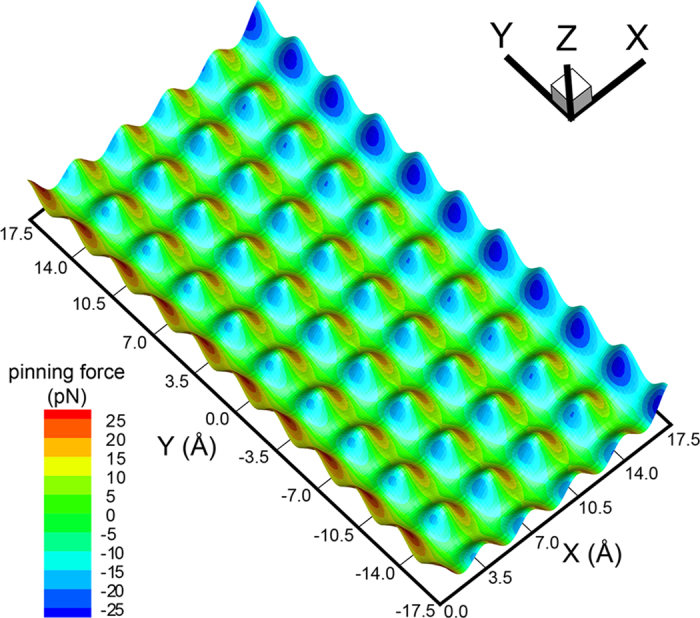
Potential energy and tangential force landscape at the top of a solid pillar. The results were obtained by using a single liquid atom located in the first liquid layer (*H* = *a*) to feel the liquid-solid interactions with the pillar. The height along Z axis represents the depth of potential energy and the magnitude of the tangential force is displayed by different colors, as shown in the color bar.

To gain further insight concerning pinning force, the next step was to consider the microscopic interactions between a single solid pillar and a small amount of liquid molecules which are used to simulate the liquid at the contact line. Figure 5 illustrates the calculated potential energy landscape felt by a single liquid atom located within the first liquid layer on top of the solid pillar. For this calculation the surface at *H* = *a* above the top of the pillar was meshed with a 0.05*a* sized grid, and the potential energy was calculated by placing a liquid “test” atom on each grid. The tangential force of the atom on each grid was also calculated. The undulation of the landscape seen in Fig. 5 indicates that the potential energy of the test atom varies across different positions. The height of the potential energy well at the edges of the pillar (along the X direction) is larger than that calculated for the center area. This is due to the fact that symmetry breads down at the edges of the pillar. In Fig. 5, the various colors used on the potential energy map represent the magnitude of the tangential force at a particular localization. The tangential force pointing toward the positive X axis has a positive value. The local extremum of the tangential force is located on the ridge of the potential energy landscape, about 0.2*a* distant from each nearest unit cell boundary. If the liquid atom did not experience any external forces, it would simply remain in the potential well and feel zero tangential force from the pillar. Otherwise, the depinning force would pull the liquid atom away from its equilibrium position and then the tangential force comes into effect. For the liquid test atom, the tangential force it experiences is the pinning force exerted by the solid pillar. This pulling experienced by the atom is a microscopic interpretation of the pinning force. Figure 5 clearly shows that a liquid atom located at different position would endure different pinning force.

**Figure 6:**
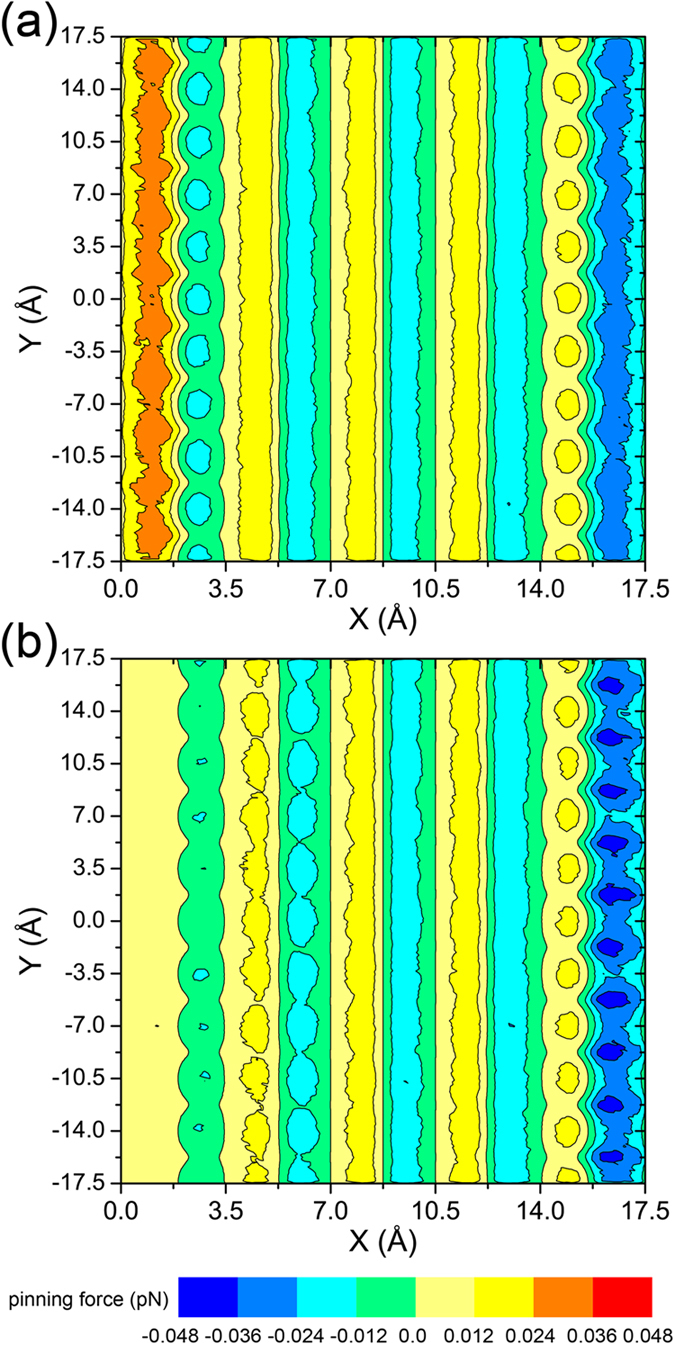
Pinning force distributions for the liquid layer located directly on top of a solid pillar. (**a**) The liquid did not experience any external forces, therefore the pinning force profile was antisymmetric, and the total pinning force on the first liquid layer was nearly zero. (**b**) A body force was applied to the liquid along the positive X axis and lead to changes in the pinning force, more obviously near the two edges.

Next, two different states of this system were compared. In the first case, the liquid on top of the pillar did not experience any extra force, and therefore was in an equilibrium state and had a symmetrical shape. In the second case, a body force was applied to the droplet along the horizontal direction to simulate the depinning force. It should be noted that in both cases, the contact line of the small droplet remains pinned on the pillar. Figure 6 shows the effects of this body force on the distribution of pinning forces at the liquid-solid interface. As in Fig. 5, the surface above the solid pillar was meshed with a 0.05*a* sized grid. The distribution profiles were obtained by multiplying the pinning force (per atom) at each grid site by the number of liquid atoms within that grid over an average of 10 ns. The magnitude of the local pinning force exerted on each liquid molecule is dependent on its specific location. It was also calculated that the liquid molecules located within the first liquid layer on top of the pillar experience the majority of the depinning force. In Fig. 6(a), the total pinning force is almost zero due to the pinning force profile being antisymmetric. Near the edges of the pillar, local pinning forces push toward the interior of the liquid, making the liquid stay on top of the pillar instead of spreading along vertical sides. In the second case, the applied force results in a tilted shape of the liquid droplet while the contact line remains pinned on the pillar. This external force also distorts the pinning force profile, particularly at the edges of the pillar, as shown in Fig. 6(b). Not only the high pinning force area near the edge of pillar, but also the center part on top of the pillar, contribute to the magnitude of the pinning force. As mentioned previously, the liquid molecules experiencing a depinning force could adjust their positions to balance out this force. Therefore, as the depinning force works to pull the liquid off the pillar, the liquid molecules move to the position where the local pinning force is larger. These results further confirmed that in the stick stage of the evaporation process, the instantaneous variation of the pinning force on the contact line is achieved by self-adaptation of the solid-liquid intermolecular distance.

The total pinning force exerted on the contact line is determined by two factors, the liquid-solid interactions, which determine the magnitude of the local pinning force and the liquid-liquid interactions, which affect the liquid distribution on top of the solid pillar. In Fig. 2(b) it was noted that during evaporation the stick-slip events occurred only when the tangential force exerted by the solid pillar reached a threshold of about 150 pN. This value indicates that the pinning force has a maximum value *f*_*pm*_ during evaporation. When the depinning force *f*_*d*_ is greater than *f*_*pm*_, the contact line is no longer pinned and starts to move. In our MD simulations, the maximum tangential force exerted by each solid pillar, *f*_*Sm*_, was initially calculated by averaging the peak values of the force curves, then *f*_*pm*_ was calculated using equation (3),


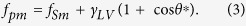


Alternatively, the maximum pinning force could also be expressed as,


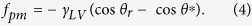


**Figure 7:**
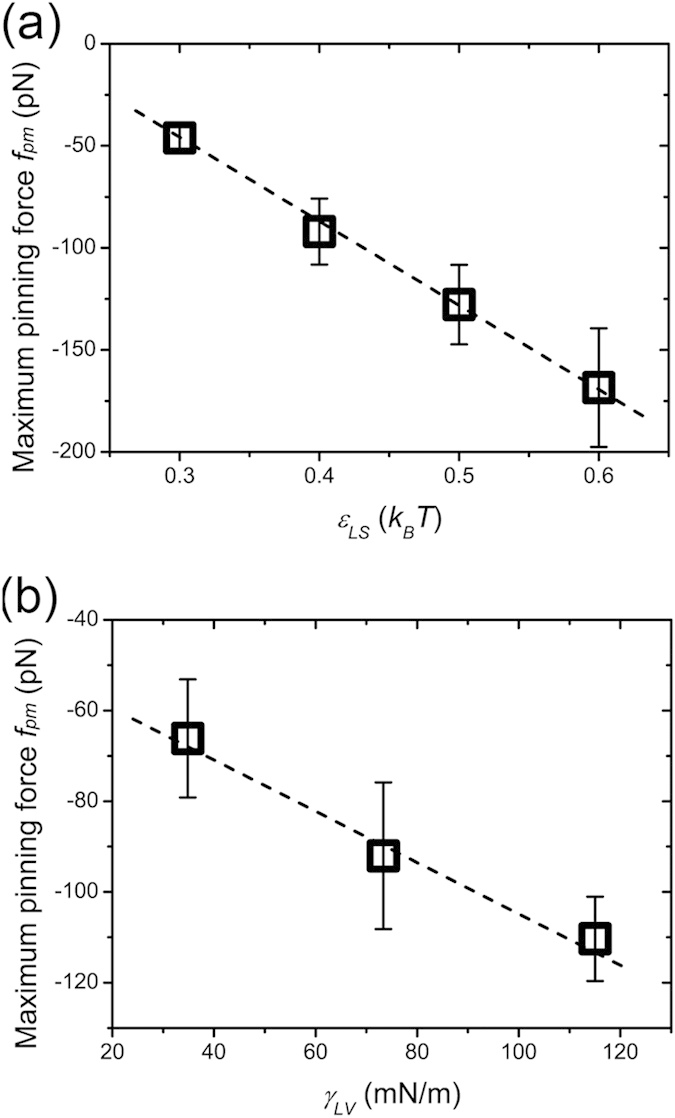
Relationships between the maximum pinning force and two influential parameters: (**a**) solid-liquid adhesion strength and (**b**) liquid-vapor surface tension. Dashed lines show linear fits. The error bars are the standard deviations.

Next, a series of evaporation simulations were completed using different *α* and *β* values to calculate *f*_*pm*_. The MD results for these simulations are summarized in Fig. 7. The results clearly demonstrate that the magnitude of *f*_*pm*_ increases linearly with the strength of solid-liquid adhesion, plotted below as an energy scale, *ε*_*SL*_. Moreover, as *γ*_*LV*_ increases, the magnitude of *f*_*pm*_ also increases. These results align well with our previous analysis and discussion. Increasing *ε*_*SL*_ results in a greater ability for the solid pillar to pin a contact line to its surface^42^. Also, the number of liquid atoms in the first liquid layer increases with increasing *ε*_*SL*_. The MD simulations show that the value of *γ*_*LV*_ increases with an increase in liquid-liquid interactions, leading to a larger number of liquid atoms being exposed to the pinning force. The solid pillar is therefore able to apply a larger pinning force on these liquid atoms and the magnitude of the *f*_*pm*_ on the contact line increases.

## Conclusions

In summary, the molecular origins of the contact line stick-slip motion observed during evaporation have been demonstrated. Based on MD simulation results, we explained how the stick-slip events take place. It is found that the pinning force on the contact line gradually increases as decreasing of the contact angle during evaporation until it reaches a maximum value and the contact line starts to slip. The analysis and simulation results illustrated that variation of the pinning force throughout the stick-slip event is accomplished by self-adaptation of the solid-liquid intermolecular distance. MD simulations also showed that the maximum pinning forces exerted on the contact line can be quantitatively assessed, and are related to both liquid-vapor surface tension and solid-liquid adhesion strength. These results are of undeniably important for understanding the essential kinetics of the contact line. In addition, these findings shed light on fundamental topics of interest to the wetting and interface science community, such as contact angle hysteresis, wetting transitions, and dynamic spreading.

## Methods

MD simulations of droplet evaporation on flexible pillared substrates were carried out using LAMMPS^43^. The simulated liquid droplet was given a diameter of about 36.7 nm and contained 10000 chain-like molecules, with each chain represented by 8 monomers attached by springs. The Lennard-Jones (LJ) potential was used to describe the interactions between non-bonded monomers as well as interactions between the liquid droplet and the pillared substrate.


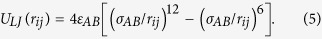


Here *A* and *B* designate different phases, such as solid (*S*), liquid (*L*), or vapor (*V*). For these simulations, *σ*_*LL*_ = *σ*_*SL*_ = 0.35 nm, 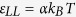, and 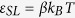, in which *k*_*B*_ is the Boltzmann constant, and *T* is the absolute temperature. *α* and *β* are coupling parameters used to control the liquid-vapor surface tension *γ*_*LV*_ and the substrate wettabilities^39^. *r*_*ij*_ gives the distance between any pair of atoms *i* and *j* with the cutoff set to 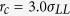. The bond between liquid monomers is described by the finite extensible nonlinear elastic potential,


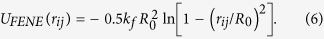


Here 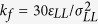 and 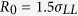
44. This chain-like model has been widely used in the literatures to investigate the wetting behaviors of nanodroplets^39^. Compared with the simple LJ particles, the viscosity of the liquid can be increased to more realistic values. On the other hand, the long-range Coulombic interactions do not need to be calculated in this model compared with water models, which can save much computing time. Thus we can study larger droplet evaporation on the pillared surfaces. We should notice that chain length and stiffness in this model would also contribute to the liquid properties, such as liquid density and surface tension, which may further influence on the contact line motion.

The solid substrate consists of regular nanometer-sized pillars of width *w* = 5*a*, height *h* = 15*a*, and interspace *l* = 5*a*, and a periodicity *d* = *w *+ *l*. Solid atoms were placed on a body-centered cubic lattice with harmonic springs connecting each atom to its nearest and next-nearest neighbors^42^. The lattice spacing was set to 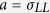, with the spring constant is 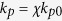, in which 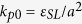 and 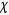 is altered to control the substrate stiffness. This is a quasi-2D model and the thickness of the simulation box was 10*a*. The droplets involved in this present work are long cylindrical cap-shaped due to the periodic boundary condition applied in this direction. It has been previously reported that cylindrical droplets are not affected by the line tension^45^,^46^.

All MD simulations were performed in the canonical ensemble with a time step of 1 fs. The temperature was set to *T* = 298 K using a Nosé-Hoover thermostat. Since the number of atoms contributing to the temperature was varying during evaporation, it is recomputed each time when the temperature is computed. After the droplet had achieved its equilibrium configuration, it was brought to wet the pillared substrate and formed a shape with an apparent contact angle θ^*^. To calculate apparent contact angle θ^*^ and the instantaneous contact angle *θ*, the droplet profiles were fitted with a circle by processing snapshots from MD simulations. Then we can obtain the base diameter and the contact angle of the droplet. Liquid molecules were then randomly and regularly deleted from the droplet to simulate evaporation, in a manner similar to our previous work^27^. The evaporation rate was set as a constant. Because its value (about 4.15 × 10^−13^ mol/s) is several orders of magnitude smaller than that observed during common macroscopic evaporation processes, our evaporation simulation is considered a quasi-static process.

In this work, the contact line was considered as the liquid corner in the vicinity of the outer border of the liquid-solid contact area. In our MD simulations, we first defined a region of thickness *a* on top of the pillared surface. Then the outer border of the liquid-solid contact area was determined by calculating the minimum and maximum of the coordinate for all liquid atoms in this area. The liquid corner starts from this outer border and has a radius equal to the cutoff used in MD simulations. This liquid corner was defined as the contact line.

## Additional Information

**How to cite this article**: Wang, F.C. and Wu, H.A. Molecular origin of contact line stick-slip motion during droplet evaporation. *Sci. Rep.* **5**, 17521; doi: 10.1038/srep17521 (2015).

## Figures and Tables

**Figure 1:**
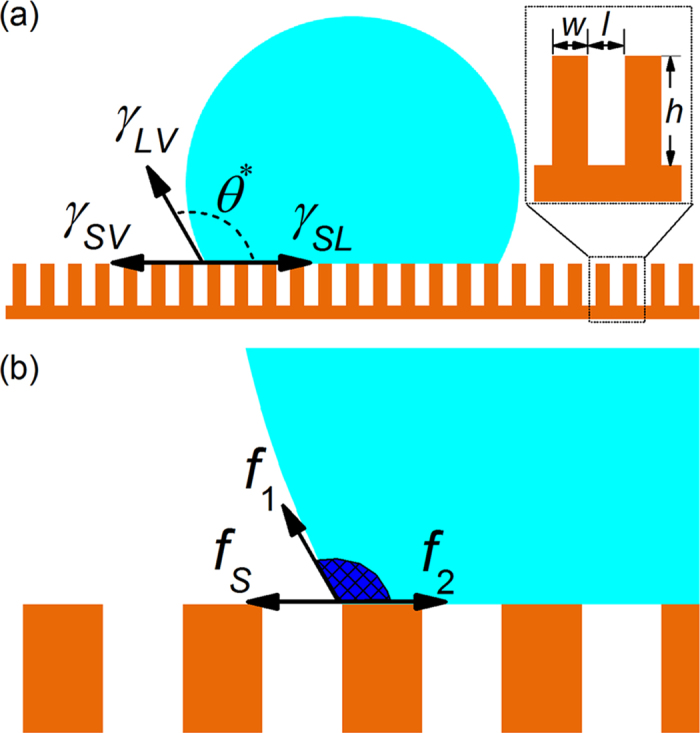
(**a**) Schematic illustration of a sessile droplet on the pillared substrate. An enlarged view of the topology of the pillared substrate is shown in the dashed box. (**b**) Microscopic perspective of the forces acting on the contact line. The meshed region indicates the liquid corner in the vicinity of the contact line.
